# Distinct Mechanisms of Pathogenic DJ-1 Mutations in Mitochondrial Quality Control

**DOI:** 10.3389/fnmol.2018.00068

**Published:** 2018-03-15

**Authors:** Daniela Strobbe, Alexis A. Robinson, Kirsten Harvey, Lara Rossi, Caterina Ferraina, Valerio de Biase, Carlo Rodolfo, Robert J. Harvey, Michelangelo Campanella

**Affiliations:** ^1^Department of Biology, University of Rome Tor Vergata, Rome, Italy; ^2^Regina Elena National Cancer Institute, Rome, Italy; ^3^Department of Pharmacology, UCL School of Pharmacy, University College London, London, United Kingdom; ^4^Department of Comparative Biomedical Sciences, Royal Veterinary College, University of London, London, United Kingdom; ^5^School of Health and Sport Sciences, University of the Sunshine Coast, Sippy Downs, QLD, Australia; ^6^Sunshine Coast Health Institute, Birtinya, QLD, Australia; ^7^UCL Consortium for Mitochondrial Research, University College London, London, United Kingdom

**Keywords:** mitochondria, mitophagy, DJ-1, DJBP/EFCAB6, *PARK7*, SUMO-1

## Abstract

The deglycase and chaperone protein DJ-1 is pivotal for cellular oxidative stress responses and mitochondrial quality control. Mutations in *PARK7*, encoding DJ-1, are associated with early-onset familial Parkinson’s disease and lead to pathological oxidative stress and/or disrupted protein degradation by the proteasome. The aim of this study was to gain insights into the pathogenic mechanisms of selected DJ-1 missense mutations, by characterizing protein–protein interactions, core parameters of mitochondrial function, quality control regulation via autophagy, and cellular death following dopamine accumulation. We report that the DJ-1^M26I^ mutant influences DJ-1 interactions with SUMO-1, in turn enhancing removal of mitochondria and conferring increased cellular susceptibility to dopamine toxicity. By contrast, the DJ-1^D149A^ mutant does not influence mitophagy, but instead impairs Ca^2+^ dynamics and free radical homeostasis by disrupting DJ-1 interactions with a mitochondrial accessory protein known as DJ-1-binding protein (DJBP/EFCAB6). Thus, individual DJ-1 mutations have different effects on mitochondrial function and quality control, implying mutation-specific pathomechanisms converging on impaired mitochondrial homeostasis.

## Introduction

Mitochondria are intimately involved in the pathogenic mechanisms underlying neurodegenerative diseases. Deficits in mitochondrial oxidative phosphorylation activity, which is the principal source of cellular energy in neurons, lead to reduced cellular viability (Schon et al., 2012). Mitochondria are one of the main sites of reactive oxygen species (ROS) production (Muller et al., 2003; Murphy, 2009), which when dysregulated leads to damage of mitochondrial components (Adam-Vizi and Chinopoulos, 2006) and functions (D’Autréaux and Toledano, 2007) contributing to the onset of neurodegeneration (Onyango et al., 2010). Equally, non-physiological changes in the overall mitochondrial mass reflect an altered balance between mitochondrial biogenesis and degradation, achieved by a specialized form of autophagy, known as mitophagy (Boland et al., 2013). Defects in any of the above-mentioned mitochondrial pathways can lead to the development and/or progression of neurodegenerative disorders such as Parkinson’s disease (PD) (Parker et al., 1989; Narendra and Youle, 2012). PD is a progressive neurodegenerative disorder (Pankratz et al., 2009) caused by the degeneration of dopaminergic neurons in the substantia nigra. Histopathologically, PD is characterized by the formation of fibrillar cytoplasmic inclusion, known as Lewy bodies, containing ubiquitin and α-synuclein, that are found in the substantia nigra as well as cortical and limbic structures (Kim et al., 2014). Even though the most frequent types of PD are idiopathic in nature (Cannon and Greenamyre, 2011; Giordano et al., 2012), around 10% of familial cases are caused by mutations in “*PARK*” genes: e.g., *PARK1/4/SNCA*, *PARK2/PARKIN*, *PARK6/PINK1* and *PARK7/DJ-1*, and *PARK8/LRRK2* (Polymeropoulos et al., 1997; Lücking et al., 2000; Abou-Sleiman et al., 2003; Paisán-Ruíz et al., 2004; Valente et al., 2004).

Mutations in *PARK7*, which encodes the protein deglycase DJ-1, cause early-onset recessive Parkinsonism (Bonifati et al., 2003). DJ-1 is a multifunctional, evolutionary-conserved protein that is ubiquitously expressed in human tissues, with highest levels in the testis and the brain (Bonifati et al., 2003, 2004). DJ-1 is involved in oxidative stress responses (Canet-Avilés et al., 2004; Ariga et al., 2013), anti-apoptotic signaling (Waak et al., 2009; Giaime et al., 2010), and protein quality control (Chen et al., 2010). In addition, DJ-1 is required for correct mitochondrial morphology and function, being involved in the degradation of dysfunctional mitochondria via autophagy (Wang et al., 2012). DJ-1 is mainly cytosolic but, after oxidative stress, associates with mitochondria promoting the removal of damaged organelles (Kahle et al., 2009; Wang et al., 2012). Cells with mutated DJ-1 are also unable to eliminate ROS sources, leading to cell death (Takahashi-Niki et al., 2004). Several missense mutations have been identified in DJ-1, including M26I, A104T, D149A, E163K, and L166P (Bonifati et al., 2003; Görner et al., 2004; Martinat et al., 2004; Olzmann et al., 2004; Shendelman et al., 2004; Taira et al., 2004; Blackinton et al., 2005; Hulleman et al., 2007; Lakshminarasimhan et al., 2008; Ariga et al., 2013; Maita et al., 2013; Ben-Nissan et al., 2016). The L166P mutation leads to severe destabilization and unfolding of DJ-1, resulting in a loss of DJ-1 dimerization. DJ-1^L166P^ is unable to function as a chaperone (Shendelman et al., 2004) or a protease (Olzmann et al., 2004), and is unable to protect cells from H_2_O_2_-induced cell death (Görner et al., 2004; Martinat et al., 2004; Taira et al., 2004). Other DJ-1 mutations (e.g., A104T, E163K, and D149A) appear to reduce the stability of DJ-1 without causing substantial folding defects or loss of dimerization (Lakshminarasimhan et al., 2008; Ben-Nissan et al., 2016). The M26I mutation has been reported to affect steady-state levels of DJ-1 (Blackinton et al., 2005), probably by increasing turnover rates and/or decreasing dimerization (Blackinton et al., 2005; Hulleman et al., 2007), thus abrogating the anti-oxidative stress function of DJ-1 (Ariga et al., 2013; Maita et al., 2013).

DJ-1 interacts with several proteins linked to oxidative stress, proteolysis, SUMOlation, and cell death, such as E3 SUMO-protein ligase PIAS2α (Takahashi et al., 2001), death-associated protein 6 (Daxx; Junn et al., 2005), Paraoxonase-2 (Parsanejad et al., 2014), and the 20S proteasome (Moscovitz et al., 2015). However, little is known about one of the first DJ-1-binding proteins (DJBP) to be identified, the DJBP (Niki et al., 2003), also known as EF-hand calcium-binding domain-containing protein 6 (EFCAB6). DJBP is known to act as a negative modulator of androgen receptor (AR) transcriptional activity (Niki et al., 2003). However, the effect of DJ-1 mutations on the DJ-1/DJBP interaction, or other binding partners, and the pathophysiological consequences DJ-1 accessory protein interactions have remained ill defined. Here, we show that several DJ-1 mutants (including A104T, D149A, and E163K) impair the DJ-1-DJBP interaction without affecting other DJ-1-accessory protein interactions or influencing the efficiency of DJ-1-dependent mitochondrial quality control. By contrast, the DJ-1 L166P mutation not only influences DJ-1 dimerization, but also disrupts interactions with all accessory proteins tested. Lastly, the DJ-1 M26I mutation abrogates the association between DJ-1 and SUMO-1, affecting mitochondrial quality control and causing increased susceptibility to dopamine-induced metabolism impairment.

## Materials and Methods

### Yeast Two-Hybrid Studies

A plasmid encoding full-length human DJ-1 fused to the LexA DNA-binding domain in the bait vector pGLex was a gift from Prof. H. Ariga (Hokkaido University, Sapporo, Japan). For prey constructs, full-length DJ-1, SUMO-1 (NM_003352.4), UBC9/UBE2I (NM_003345.4), and DJBP/EFCAB6 (AB073862) were amplified by PCR from human frontal cortex cDNA using specific oligonucleotide primers (**Supplementary Table S1**) and cloned into the pACT2 vector (Clontech) between the *Nco*I and *Xho*I sites, in-frame with the upstream GAL4 activation domain (AD) sequence. The pGAD-PIAS2α construct, encoding human full-length E3 SUMO-protein ligase PIAS2α (NP_775298.1) fused to the GAL4 AD sequence, was a gift from Prof. H. Ariga, Hokkaido University, Sapporo, Japan. A human substantia nigra cDNA library screen using pGLex-DJ-1 as bait resulted in the generation of a pGADT7Rec-HIPK2 construct; encoding amino acids 818-884 of homeodomain-interacting protein kinase 2 (HIPK2 isoform 2; NP_001106710.1) fused to the GAL4 AD sequence. A human frontal cortex cDNA library screen using pGLex-DJ-1 as bait resulted in the generation of a pGADT7Rec-SIMC1 construct, encoding amino acids 1-245 of SIMC1 (SUMO interacting motifs containing 1, also known as PLEIAD/C5orf25; Ono et al., 2013) fused to the GAL4 AD sequence. Full-length Daxx (Junn et al., 2005; NM_001350.4) was amplified using IMAGE clone IMAGp998C115401Q (Source Bioscience) and specific oligonucleotide primers (**Supplementary Table S1**) and cloned into the *Bam*HI and *Xho*I sites of pACT2. The two-hybrid assay was performed with *Saccharomyces cerevisiae* L40 cells transformed with the bait plasmid pGLex-DJ-1 [wild-type (wt) or mutant] and a prey plasmid expressing GAL4 AD fused to the indicated protein. All transformants were plated on selective medium lacking leucine and tryptophan and the resulting colonies were tested for β-galactosidase activity by performing a *LacZ* freeze-fracture assay as previously described (Harvey et al., 2004).

### Mammalian Expression Constructs

A full-length FLAG-tagged DJ-1 sequence was cloned into the *Kpn*I and *Xho*I sites of the pcDNA3.1+ vector to generate pcDNA3-FLAG-DJ-1. The DJBP cDNA encoding the 570 amino acid isoform (AB073862) was amplified from human whole-brain cDNA (Clontech) using specific oligonucleotide primers (**Supplementary Table S1**) and cloned into the *Bam*HI and *Xba*I sites of pRK5myc to generate pRK5myc-DJBP(570). Sequences encoding amino acids 1–31 and 1–48 of full-length DJBP/EFCAB6 (NM_022785.3) were amplified from a DKFZ clone (DKFZp686C1630Q; RZPD German Resource Center for Genome Research) using specific oligonucleotide primers (**Supplementary Table S1**) and cloned into the *Xho*I and *Bam*HI sites of pEGFP-N1 (Clontech) in-frame with EGFP. The mitochondrial targeting sequence from human cytochrome c oxidase subunit VIII A (NM_004074.2) was amplified from human frontal cortex cDNA (Clontech) using specific oligonucleotide primers (**Supplementary Table S1**). An *Eco*RI/*Bam*HI fragment was cloned into the MCS of pDsRed1-N1 (Clontech) to generate pDsRed1-MTS. DJ-1 missense mutations were introduced into plasmid constructs by PCR-mediated site-directed mutagenesis (QuikChange, Agilent) using pairs of complementary oligonucleotide primers (**Supplementary Table S2**).

### DJ-1/DJBP Interactions

HEK293T cells were cultured in Dulbecco’s modified Eagles medium (Invitrogen) supplemented with 10% (v/v) fetal bovine serum (FBS), 100 units/ml penicillin, and 100 μg/ml streptomycin. Cells were transiently transfected using Effectene (Qiagen) according to the manufacturers’ instructions. All stages in protein extraction and the subsequent co-purification procedure were performed at 4°C; 24 h post-transfection the cells were washed in phosphate-buffered saline (PBS) and lysed in 1% (v/v) Nonidet P-40 buffer (150 μM NaCl, 10 μM Tris–HCl, pH 8, 1× complete protease inhibitor cocktail, in PBS) for 1 h. Cell extracts were cleared by centrifugation at 3,000 × *g* for 20 min. The protein concentration of the lysate samples was determined using the Quick Start Bradford Dye Reagent (BioRad). For co-purification of FLAG-tagged fusion proteins and associated proteins, lysates were incubated with anti-FLAG agarose (Sigma) for 2 h. The bound agarose was washed three times with 0.1% (v/v) Nonidet P-40 wash buffer (150 μM NaCl, 10 μM Tris–HCl; pH 7.8) before eluting the bound FLAG protein complex by the addition of FLAG peptide (Sigma). Co-purification or total lysate samples were resolved by 10% sodium dodecyl sulfate (SDS)–polyacrylamide gel electrophoresis (PAGE) and subjected to Western blot analysis with mouse monoclonal anti-FLAG antibody (M2; Sigma), mouse monoclonal anti-c-myc antibody (9E11; Abcam), mouse monoclonal anti-DJ-1 antibody (3E8; Stressgen), rabbit polyclonal anti-DJ-1 antibody (No. 2134; New England Biolabs), or mouse monoclonal anti-α-tubulin (DM 1A; Sigma). Bands were visualized by enhanced chemiluminescence (Amersham Biosciences).

### DsRed-Mito Localization

HEK293 cells were seeded in 40 mm TC dishes containing a poly-L-lysine-coated coverslip and co-transfected with 0.25 μg pDsRed-Mito and 0.75 μg pEGFP-DJBP constructs. Twenty-four hours post-transfection the cells were fixed with 4% (w/v) paraformaldehyde and quenched with 50 mM NH_4_Cl. The coverslip was mounted and the slides were examined using the Zeiss LSM 510 Microscope equipped with argon (excitation 488 nm) and helium-neon (543 nm and 633 nm) lasers.

### SH-SH5Y Cell Culture and Transfection

The human neuroblastoma cell line SH-SY5Y purchased from American Type Culture Collection (ATCC, Rockville, MD, United States) was grown in Dulbecco’s modified eagles medium:Nutrient Mixture F-12 (DMEM-F12) (Sigma-Aldrich), supplemented with 10% (v/v) FBS (Lonza), 100 U/ml penicillin, and 100 mg/ml streptomycin (Life Technologies) at 37°C and 5% CO_2_. Cells were transiently transfected using CaCl_2_. On the day before transfection, cells were split in order to have an appropriate confluence (70%). The next day, medium was replaced on each plate with fresh medium at least 1 h before transfection. Later 6 μg of vector DNA (half the quantities of each for co-transfection), 2 M CaCl_2_, and H_2_O_2_ were mixed in 1× HBS. The solution was then added directly to the plate and incubated at 37°C, 5% CO_2_, for 16–24 h. After incubation, the transfection medium was replaced with fresh warm DMEM F12 for 24 h. The pcDNA3.1 (+) FLAG vector was used for over-expressing wt, M26I, and D149A DJ-1 cDNAs. Successful expression of FLAG-DJ-1 proteins was verified by Western blotting.

### Cellular Survival Assay

Cell survival was measured using 3-(4,5-dimethylthiazol-2-yl)-2,5-diphenyltetrazolium bromide (MTT) assays to determine mitochondrial activity. SH-SY5Y cell lines over-expressing wt and mutant DJ-1 were plated (50,000 cells per well, in duplicate) in 24-well plates 3 days before treatment. In particular, 48 h after transfection, cells were treated with dopamine 300 μM for 24 h. After treatment, cell medium was removed and cells were incubated in 0.5 mg/ml MTT solution for 1 h at 37°C and 5% CO_2_. To stop incubation, MTT solution was removed and dimethyl sulfoxide (DMSO) was added to solubilize the formazan product. The absorbance was monitored at 595 nm with Varioskan^TM^ LUX Multimode Microplate Reader (Thermo Fisher Scientific). The data are expressed as ratio of viable cells normalized to non-treated controls.

### Cell Death Analysis

To assay cell death in SH-SY5Y over-expressing wt and mutant DJ-1, the cells were grown on coverslips and treated with 10 μM cycloheximide *N*-acetyl-D-sphingosine (C2 ceramide) for 14 h or 100 μM H_2_O_2_ for 8 h. After incubation, cells were washed thoroughly to eliminate apoptotic cells. The percentage of fluorescent cells before and after a challenge with the apoptotic drugs was determined *de visu* by counting at the microscope.

### Aequorin Measurements

For the mtAEQ and cytAEQ measurements, the cells, over-expressing wt and mutant DJ-1, in presence or absence of H_2_O_2_ (2 h, 100 μM) were incubated with 5 μM coelenterazine for 1–2 h in DMEM supplemented with 1% FBS and then transferred to the perfusion chamber as previously reported (Campanella et al., 2004). The aequorin luminescence data were calibrated off-line into [Ca^2+^] values, using a computer algorithm based on the Ca^2+^ response curve of wt and mutant aequorins.

### Western Blotting Analysis

After treatment with different reagents including CCCP (4–8 h, 10 μM), dopamine (24 h, 100–300 μM), and chloroquine (CLQ) (8 h, 1:1000), SH-SY5Y cells transiently transfected with wt and mutant DJ-1 constructs were washed in PBS and collected by gentle scraping and incubated in ice-cold RIPA buffer [50 mM Tris–HCl, 150 mM NaCl, 1% (v/v) Triton-X100, pH 8.0] containing protease and the phosphatase inhibitor for 30 min on ice. Lysates were centrifuged at 13,000 × *g* at 4°C for 20 min. For subcellular fractionation, cells were collected in fractionation buffer (250 mM sucrose, 20 mM HEPES, 10 mM KCl, 1.5 mM MgCl_2_, 1 mM Na_2_-EDTA, 1 mM EGTA, pH to 7.4, supplemented with protease inhibitors, just prior to use). Cell suspensions were passed through a 26G needle 50–80× and then centrifuged at 900 × *g* for 5 min at 4°C. The supernatant was collected and centrifuged again at 10,000 × *g* for 10 min at 4°C. The resulting pellet, containing a fraction enriched in mitochondria, was suspended in fractionation buffer and after another centrifugation at 10,000 × *g* for 5 min at 4°C, suspended in standard lysis buffer [50 mM Tris, pH 8.0, 150 mM NaCl, 1% (v/v) Triton-X100] supplemented with protease inhibitors. After 30 min incubation on ice, samples were centrifuged for 20 min at 130,000 × *g*. Protein concentration was estimated using the Bradford reagent, and 20 μg of total proteins was mixed with Laemmli sample buffer and boiled at 95°C for 2 min. Proteins were resolved by SDS–PAGE and transferred to nitrocellulose (mdi, Membrane Technologies) or PVDF (Millipore) membranes. Membranes were blocked in 5% non-fat dry milk (following manufacturer recommendation) in 1× TBST (25 mM Tris, 0.15 M NaCl, 0.05% Tween-20, pH 7.5) for 1 h, and then probed with appropriate diluted primary antibodies in 5% non-fat dry milk overnight at 4°C. Antibodies used were: mouse monoclonal anti-p62 1 μg/ml (MBL); rabbit polyclonal anti-VDAC1 1 μg/ml (Abcam); rabbit polyclonal anti-LC3 0.5 μg/ml (Novus Biologicals); mouse monoclonal anti-GAPDH 0.2 μg/ml (Abcam); rabbit polyclonal anti-ubiquitin at 0.5 mg/ml (Abcam), rabbit polyclonal anti-PINK1 at 2 μg/ml (Novus Biologicals); mouse monoclonal anti-Parkin at 2 μg/ml (Sigma–Aldrich); rabbit polyclonal anti-ATPβ at 1 μg/ml (Abcam); goat polyclonal anti-DJBP at 0.4 μg/ml (Santa Cruz); rabbit polyclonal anti-DJ-1 at 0.265 μg/ml (Abcam); rabbit polyclonal anti-PARP at 0.5 μg/ml (Abcam); mouse monoclonal anti-MTCO1 at 1 μg/ml (Abcam); rabbit polyclonal anti-β_III_ tubulin at 1 μg/ml (Abcam); and rabbit polyclonal SUMO-1 1 μg/ml (Cell Signaling). Following incubation, membranes were washed in 1× TBST (3 × 15 min at RT) and then probed with the corresponding peroxidase-conjugated secondary antibody in 5% non-fat milk for 1 h at RT. After further washing in 1× TBST, blots were developed using an ECL detection kit (Millipore) and a FluorChem imaging system. Immunoreactive bands were analyzed by performing densitometry analysis with ImageJ software (NIH, Bethesda, MD, United States).

### Immunofluorescence and Image Analysis

Cells transiently transfected with wt and mutant DJ-1 were cultured on poly-D-lysine glass coverslips (24Ø) for 24 h. After administration of CCCP 10 μM for 8 h, cells were washed twice in cold PBS (pH 7.4) and fixed in 4% (w/v) paraformaldehyde for 20 min at RT. Following three washes in PBS, cells were permeabilized with 0.1% Triton-X100 (AppliChem PanReac) for 10 min at RT and blocked with 3% (w/v) bovine serum albumin for 1 h at RT. All coverslips were then incubated with primary antibodies (goat polyclonal anti-DJBP at 0.4 μg/ml, Santa Cruz) and rabbit polyclonal anti-ATPβ at 1 μg/ml (Abcam) at 4°C overnight. Following three washes in PBS, coverslips were incubated in the dark with a mix of the following secondary antibodies AlexaFluor 594-conjugated anti-goat IgG and AlexaFluor 488-conjugated anti-rabbit IgG (Invitrogen) at 1:100 for 1 h at RT. After a final wash, coverslips were mounted with ProLong Gold antifade reagent (Invitrogen) containing DAPI (Applichem), for nuclei visualization, and imaged by means of an Olympus Fluoview 1000 Confocal Laser Scanning System. All images were analyzed by ImageJ software (NIH, Bethesda, MD, United States).

### Flow Cytometric Analyses

SH-SY5Y cell lines transiently transfected with wt and DJ-1 mutants were treated with 100 μM H_2_O_2_ for 20 min or 300 μM dopamine for 24 h, washed with PBS, harvested with trypsin, and suspended in warm DMEM F12 containing the appropriate fluorescent dye. The mitochondrial membrane potential was assessed upon staining with 1 μM JC-1 (Invitrogen) for 10 min at 37°C; mitochondria generated ROS by staining with 5 μM MitoSOX (Invitrogen) for 10 min at 37°C. Samples were collected by means of a BD FACSCalibur and data analyzed with FlowJo (TreeStar).

### Statistical Analysis

Statistical analyses were performed by means of two-way ANOVA, followed by Bonferroni test for multiple comparisons with GraphPad Prism 5. For the immunofluorescence data Pearson’s/Mander’s correlation test was used. Statistical significance was accepted at the 95% confidence level (^∗∗∗^*p* < 0.001; ^∗∗^0.001 < *p* < 0.01; ^∗^0.01 < *p* < 0.05).

## Results

### Mutated DJ-1 Isoforms and Their Interaction With Accessory Proteins

**FIGURE 1:**
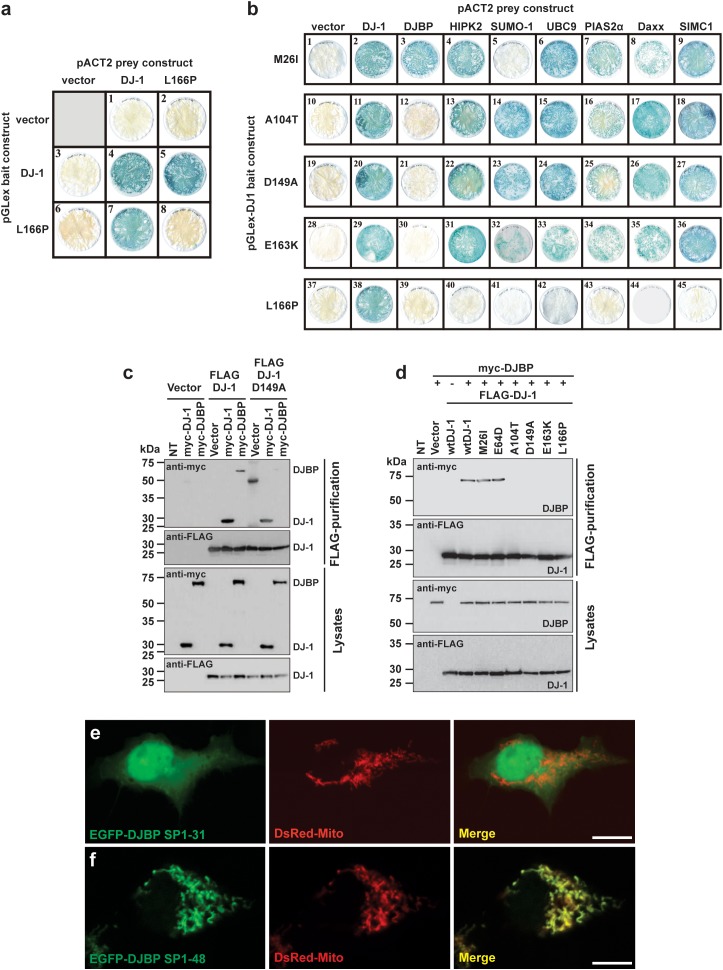
Different DJ-1 mutants disrupt interactions with either SUMO-1 or DJBP. **(a)** YTH β-galactosidase filter assays measuring the effect of the DJ-1^L166P^ mutation on DJ-1–DJ-1 dimerization. Representative filters for β-galactosidase activity assay are shown. Blue-colored colonies indicate the detection of protein–protein interactions. **(b)** The effect of DJ-1 missense mutations on interactions with binding partners. Mutant DJ-1 baits, indicated on the left side of the figure, were tested against the accessory protein preys indicated at the top of the figure. Note that mutation M26I disrupts interactions with SUMO-1, A104T, D149A, and E163K specifically disrupt DJ-1/DJBP interactions and L166P disrupts all DJ-1 accessory protein interactions. **(c)** Affinity purification and immunoblotting analyses of HEK293 cell lysates demonstrating that the DJ-1^D149A^ mutant does not influence DJ-1/DJ-1 interactions, but disrupt DJ-1/DJBP interactions. **(d)** Affinity purification and immunoblotting analyses of HEK293 cell lysates showing that mutations A104T, D149A, E163K, and L166P also disrupt DJ-1/DJBP interactions in cellular assays, while retaining DJ-1–DJ-1 interactions. **(e,f)** Confocal imaging of the subcellular localization of DJBP signal peptide–EGFP fusion proteins EGFP–DJBP SP1-31 **(e)** and EGFP–DJBP SP1-48 **(f)**, co-transfected with mitochondrial marker (DsRed-Mito). EGFP–DJBP SP1-31 shows cytoplasmic and nuclear localization, while EGFP–DJBP SP1-48 is targeted to mitochondria, as shown in the merged images. Scale bar = 10 μm.

The effect of reported DJ-1 missense variants M26I, E64D, R98Q, A104T, K130R, D149A, E163K, and L166P on the formation of DJ-1 dimers was assessed using the yeast two-hybrid (YTH) system (**Figure 1a** and **Supplementary Figure S1a**). Each of the mutant pGLex-DJ-1 bait constructs was tested for interaction against the corresponding mutant pACT2-DJ-1 prey constructs (**Supplementary Figure S1a**). All of the DJ-1 mutants could form heterodimers with wt DJ-1, including L166P (**Figure 1a**). However, DJ-1^L166P^ was incapable of homodimer formation (**Figure 1a** and **Supplementary Figure S1a**). These results support the YTH study of Takahashi-Niki et al. (2004) and suggest that disruption of DJ-1 dimerization is not a common pathogenic mechanism underlying DJ-1-related PD (**Supplementary Figure S1a**). For this reason, DJ-1 mutants were also assessed for their ability to interact with a range of published and newly identified DJ-1 accessory proteins, using the same YTH system (**Figure 1b**). Each of the mutant pGLex-DJ-1 bait constructs was tested for interaction against Daxx, DJBP/EFCAB6, HIPK2, SUMO-1, UBC9, PIAS2α, and SIMC1/PLEIAD/C5orf25 prey constructs. Interestingly, the M26I mutation was found to specifically disrupt the interaction between DJ-1 and SUMO-1 (**Figure 1b**). This is likely to be functionally relevant, because incorrectly SUMOylated DJ-1 becomes insoluble, partly localized in the mitochondria, and degraded by the proteasome system (Shinbo et al., 2006). By contrast, the A104T, D149A, and E163K mutations specifically disrupted interactions between DJ-1 and DJBP. It is also notable that the L166P mutation abolished all DJ-1-accessory protein interactions, suggesting that DJ-1 dimerization is required for DJ-1 to interact with these accessory proteins. Since all other protein–protein interactions (with Daxx, HIPK2, PIAS2α, SIMC1/PLEIAD/C5orf25, and UBC9) were unaffected, this means that at least four pathogenic variants in DJ-1 appear to operate by disrupting DJ-1/DJBP interactions.

To confirm these findings in a non-yeast system, we tested these protein–protein interactions with an anti-FLAG affinity purification procedure, in a mammalian cell expression system (HEK293T cells). Anti-FLAG affinity resin was used to pull down N-terminal FLAG-tagged wt and mutant DJ-1 (FLAG-DJ-1), over-expressed in combination with N-terminal myc-tagged DJBP (myc-DJBP) (**Supplementary Figure S1a**). As in the YTH system, the DJ-1 missense mutations A104T, D149A, E163K, and L166P (but not wt, M26I, or E64D mutants) clearly disrupted the interaction between exogenously expressed FLAG-DJ-1 and myc-DJBP (**Figures 1c,d**) without affecting DJ-1–DJ-1 interactions. Currently very little is known about DJBP, with only one study describing the identification of DJBP as a DJ-1 interacting protein *in vitro* and *in vivo* (Niki et al., 2003). In that study, the DJBP cDNA was reported to consist of 2119 nucleotides, encoding a 570 amino-acid protein. We performed a nucleotide database search, with the 2119 bp cDNA DJBP sequence, which revealed the identification of additional, longer DJBP variants. The longest cDNA consisted of 4817 nucleotides, encoding a 1501 amino acid protein, and a slightly shorter 4459 nucleotide cDNA encoding a 1349 amino acid protein. The use of online tools to identify potentially functional protein domains revealed the presence of 17 EF-hand motifs in the longest DJBP isoform. The presence of numerous EF-hand motifs, which are Ca^2+^ binding domains, suggests that DJBP may function as a Ca^2+^ binding protein. Bioinformatic analysis of DJBP for subcellular localization targeting sequences led to the identification of a predicted N-terminal mitochondrial-targeting sequence in the longest isoform that was examined in more detail. A potential R-10 motif was found within the N-terminal 31 amino acids and numerous potential R-2 motifs were present within the N-terminal 48 amino acids of the longest DJBP isoform (**Supplementary Figure S1b**). To examine these predicted mitochondrial-targeting motifs experimentally, cDNAs encoding N-terminal DJBP sequence (signal peptide; SP) were cloned into an EGFP vector to generate pEGFP-DJBP SP1-31 and pEGFP-DJBP SP1-48 constructs. Each construct was used to transfect HEK293 cells and the subcellular localization of the expressed EGFP-tagged DJBP SP fusion was analyzed by confocal microscopy. To facilitate the analysis, cells were co-transfected with the mitochondrial marker DsRed-Mito. We found that EGFP-DJBP SP1-31 did not localize to the mitochondria, as green fluorescence was detected throughout the cell, including the nucleus (**Figure 1e**). However, EGFP-DJBP SP 1-48 was not uniformly distributed throughout the cell but showed a strong co-localization with DsRed-Mito (**Figure 1f**), indicating that this DJBP signal peptide could confer mitochondrial localization on EGFP. This analysis also suggests that the signal peptide cleavage site is likely to be an R-2 motif located between amino acids 32 and 48 (three sites were predicted; **Supplementary Figure S1b**). Loss of interaction between DJ-1 and DJBP could therefore underlie mitochondrial dysfunction and we therefore examined this in regard to mitochondrial Ca^2+^ signaling, morphology, and quality control by autophagy, using DJ-1^D149A^ as an example of a DJ-1 mutant that does not interact with DJBP.

### The DJ-1^D149A^ Mutation Impairs Ca^2+^ Homeostasis by Increasing Cellular Sensitivity to Oxidative Stress

**FIGURE 2:**
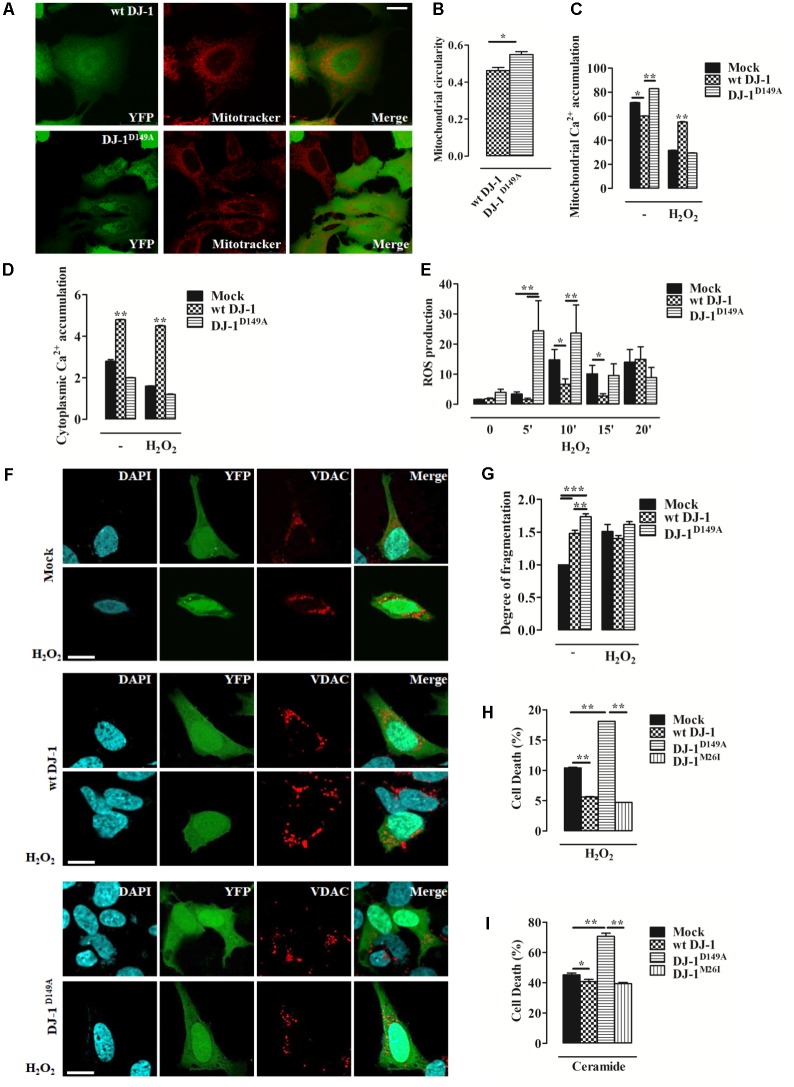
Loss of the DJ-1/DJBP interaction leads to aberrant Ca^2+^ signaling and redox-stress susceptibility. **(A)** Representative image of mitochondrial morphology in SH-SY5Y cells transiently co-transfected with FLAG-DJ-1 (wt DJ-1) or mutant FLAG-DJ-1^D149A^ (DJ-1^D149A^) together with YFP and mitoTracker red to unveil mitochondrial morphology quantified in **(B)**. [mitochondrial circularity, wt DJ-1: 0.46 ± 0.02, DJ-1^D149A^: 0.55 ± 0.02; results are shown as mean ± standard error of the mean (SEM) (*n* = 3; ^∗^0.01 < *p* < 0.05)]. Scale bar = 10 μm. **(C,D)** Peak values of mitochondrial Ca^2+^ accumulation recorded with targeted Aequorin before and after H_2_O_2_-induced redox stress (2 h, 100 μM) are presented as a histogram in panel **(C)** while data points in panel **(D)** refer to the cytoplasmic Ca^2+^ accumulation in SH-SY5Y transiently transfected with FLAG-DJ-1 (wt DJ-1) or FLAG-DJ-1^D149A^ constructs [mitochondrial Ca^2+^ accumulation, untreated Mock: 71.3 ± 0.05, wt DJ-1: 60.1 ± 0.11, DJ-1^D149A^: 82.8 ± 0.05; 2 h H_2_O_2_ 100 μM Mock: 31.7 ± 0.05, wt DJ-1: 55.1 ± 0.16, DJ-1^D149A^: 29.4 ± 0.04; cytoplasmic Ca^2+^ accumulation, untreated Mock: 2.8 ± 0.03, wt DJ-1: 4.8 ± 0.01, DJ-1^D149A^: 2.0 ± 0.01; 2 h H_2_O_2_ 100 μM Mock: 1.6 ± 0.005, wt DJ-1: 4.5 ± 0.011, DJ-1^D149A^: 1.2 ± 0.007; data are shown as mean ± SEM (*n* = 6; ^∗∗^0.001 < *p* < 0.01; ^∗^0.01 < *p* < 0.05)]. **(E)** Histogram bearing the FACS quantification of mitochondrial O^2-^ production in SH-SY5Y transiently transfected with FLAG-DJ-1 (wt DJ-1) or FLAG-DJ-1^D149A^ constructs after treatment with 100 μM H_2_O_2_ for 20 min [ROS production, untreated Mock: 1.62 ± 0.02, wt DJ-1: 1.78 ± 0.36, DJ-1^D149A^: 3.98 ± 1.04; 5 min H_2_O_2_ 100 μM Mock: 3.41 ± 0.74, wt DJ-1: 1.54 ± 0.53, DJ-1^D149A^: 24.47 ± 10.01; 10 min H_2_O_2_ 100 μM Mock: 14.81 ± 3.41, wt DJ-1: 6.70 ± 1.76, DJ-1^D149A^: 23.65 ± 9.32; 15 min H_2_O_2_ 100 μM Mock: 10.14 ± 2.80, wt DJ-1: 2.81 ± 0.77, DJ-1^D149A^: 9.56 ± 3.86; 20 min H_2_O_2_ 100 μM Mock: 14.01 ± 4.18, wt DJ-1: 14.98 ± 4.12, DJ-1^D149A^: 8.90 ± 3.33; data were shown as mean ± SEM (*n* = 3; ^∗∗^0.001 < *p* < 0.01; ^∗^0.01 < *p* < 0.05)]. **(F)** Representative images on the degree of mitochondrial fragmentation following H_2_O_2_ treatment for 20 min in SH-SY5Y cells expressing FLAG-DJ-1 (wt DJ-1) or FLAG-DJ-1^D149A^ with relative quantification reported in **(G)**. Nuclei were labeled with DAPI (blue) whereas mitochondrial morphology was detected with VDAC (red) [degree of fragmentation, untreated Mock: 1.00 ± 0.00, wt DJ-1: 1.48 ± 0.04, DJ-1^D149A^: 1.73 ± 0.05; 20 min H_2_O_2_ Mock: 1.5 ± 0.10, wt DJ-1: 1.40 ± 0.04; DJ-1^D149A^: 1.61 ± 0.04; data are shown as mean ± SEM (*n* = 3; ^∗∗∗^*p* < 0.001; ^∗∗^0.001 < *p* < 0.01)]. Scale bar = 10 μm. **(H,I)** The degree of cell death (the increase of dead cells presented as percentage and normalized to the total number of living cells) following treatment with 100 μM H_2_O_2_ for 8 h and 10 μM ceramide for 14 h in SH-SY5Y cells expressing either YFP-tagged wt DJ-1, DJ-1^D149A^, or DJ-1^M26I^ is depicted by the histograms in panels **(H)** and **(I)**. [cell death (%): 8 h H_2_O_2_ 100 μM Mock: 10.4 ± 0.05, wt DJ-1: 5.60 ± 0.04, DJ-1^D149A^: 18.10 ± 0.0015, DJ-1^M26I^: 4.7 ± 0.006; 14 h ceramide 10 μM Mock: 45.1 ± 0.7, wt DJ-1: 40.7 ± 0.8, DJ-1^D149A^: 70.60 ± 1.0, DJ-1^M26I^: 39.3 ± 0.4; data are shown as mean ± SEM (*n* = 4; ^∗∗^0.001 < *p* < 0.01; ^∗^0.01 < *p* < 0.05)].

We investigated mitochondrial network morphology in SH-SY5Y cells expressing YFP-tagged wt DJ-1 and DJ-1^D149A^ loaded with MitoTracker red (**Supplementary Figures S1c–e**). This analysis revealed tangible alterations in the overall mitochondrial network in cells expressing DJ-1^D149A^ (**Figures 2A,B**). We also assessed mitochondrial Ca^2+^ handling using the targeted luminescent probe Aequorin, which revealed differences in both the mitochondrial and cytosolic environment in DJ-1^D149A^ expressing cells. The DJ-1^D149A^ mutant dramatically increased mitochondrial Ca^2+^ accumulation compared to cells over-expressing wt DJ-1 and controls (mock transfected), while the cytosolic peaks were decreased at resting condition (**Figures 2C,D**). This suggests that the interaction of DJ-1 with DJBP has a key role in Ca^2+^ buffering. Moreover, mitochondrial fragmentation and Ca^2+^ accumulation in cells expressing DJ-1^D149A^ leads to increased ROS production (**Supplementary Figure S2a**). The same analysis, repeated following cellular treatment with H_2_O_2_ (2 h, 100 μM), normalized the differences and showed retained Ca^2+^ tonicity in cells over-expressing wt DJ-1 but not DJ-1^D149A^ (**Figures 2C,D**). This suggests that DJ-1, via interactions with DJBP, plays a homeostatic role under stress (20’ H_2_O_2_, 100 μM), as corroborated by the increase of mitochondrial generated ROS (**Figure 2E**) and mitochondrial network fragmentation already at resting condition in cells expressing DJ-1^D149A^ (**Figures 2F,G**). Interestingly, the overexpression of DJ-1^D149A^ leads to increased cell death under oxidative stress (8 h H_2_O_2_, 100 μM) (**Figure 2H**), similar to the response seen for the Ca^2+^ dependent pro-apoptotic stimulus ceramide (14 h, 10 μM) (**Figure 2I**). By contrast, the DJ-1^M26I^ mutant behaved similarly to wt DJ-1 in cells exposed to Ca^2+^ mobilizing stimuli and redox stress, suggesting that this mutant does not act via the same pathomechanism as DJ-1^D149A^.

### Effect of DJ-1^M26I^ and DJ-1^D149A^ Mutants on Mitochondrial Quality Control

**FIGURE 3:**
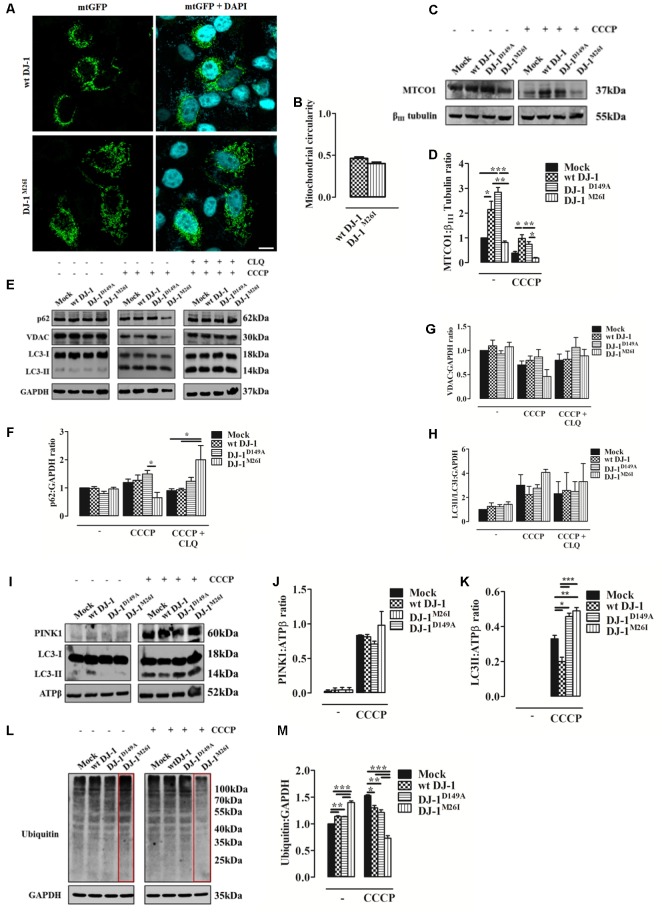
Loss of the DJ-1/SUMO interaction deregulates cellular mitophagy. **(A)** Analysis of mitochondrial morphology in SH-SY5Y cells expressing the FLAG-DJ-1^M26I^ construct with quantification in **(B)** [mitochondrial circularity, wt DJ-1: 0.46 ± 0.02, DJ-1^M26I^: 0.40 ± 0.02; results are shown as mean ± SEM (*n* = 3; no variance was observed)]. Scale bar = 10 μm. **(C)** Immunoblotting analysis of mitochondrial DNA (mtDNA) encoded protein MTCO-1 content after CCCP treatment (8 h, 10 μM) in SH-SY5Y cells co-transfected with wt FLAG-DJ-1, FLAG-DJ-1^D149A^, or FLAG-DJ-1^M26I^ constructs 24 h after transfection. Data, quantified in **(D)**, are shown as mean ± SEM (*n* = 3; ^∗∗∗^*p* < 0.001; ^∗∗^0.001 < *p* < 0.01; ^∗^0.01 < *p* < 0.05) [MTCO-1 density relative to βIII tubulin, untreated Mock: 1.00 ± 0.00, wt DJ-1: 2.15 ± 0.33, DJ-1^D149A^: 2.84 ± 0.19, DJ-1^M26I^: 0.81 ± 0.06; 8 h CCCP 10 μM Mock: 0.39 ± 0.06, wt DJ-1: 0.98 ± 0.14, DJ-1^D149A^: 0.74 ± 0.10, DJ-1^M26I^: 0.18 ± 0.02]. **(E–H)** Total extracts were then analyzed by Western blotting, testing LC3 degree of activation, p62, and VDAC before and after treatment with chloroquine (CLQ) (8 h, 1:1000) **(E)**. Data were normalized on the basis of GAPDH levels and quantified in **F–H** [p62 density, untreated Mock: 1 ± 0.00, wt DJ-1: 0.99 ± 0.06, DJ-1^D149A^: 0.80 ± 0.09, DJ-1^M26I^: 0.96 ± 0.06; 8 h CCCP 10 μM Mock: 1.19 ± 0.12, wt DJ-1: 1.27 ± 0.19, DJ-1^D149A^: 1.49 ± 0.13, DJ-1^M26I^: 0.65 ± 0.18; 8 h CCCP 10 μM + 8 h CLQ 1:1000 Mock: 0.90 ± 0.07, wt DJ-1: 0.94 ± 0.05, DJ-1^D149A^: 1.24 ± 0.14, DJ-1^M26I^: 2.00 ± 0.51; (*n* = 5; ^∗^0.01 < *p* < 0.05); VDAC density, untreated Mock: 1 ± 0.00, wt DJ-1: 1.10 ± 0.12, DJ-1^D149A^: 0.93 ± 0.07, DJ-1^M26I^: 1.08 ± 0.09; 8 h CCCP 10 μM Mock: 0.70 ± 0.08, wt DJ-1: 0.80 ± 0.09, DJ-1^D149A^: 0.87 ± 0.15, DJ-1^M26I^: 0.46 ± 0.14; 8 h CCCP 10 μM + 8 h CLQ 1:1000 Mock: 0.80 ± 0.13, wt DJ-1: 0.82 ± 0.17, DJ-1^D149A^: 1.06 ± 0.20, DJ-1^M26I^: 0.89 ± 0.13 (5 < *n* < 8, no variance was observed); LC3II/LC3I density, untreated Mock: 1 ± 0.00, wt DJ-1:1.26 ± 0.29, DJ-1^D149A^: 1.23 ± 0.18, DJ-1^M26I^: 1.43 ± 0.21; 8 h CCCP 10 μM Mock: 3.01 ± 0.87, wt DJ-1: 2.23 ± 0.69, DJ-1^D149A^: 2.76 ± 0.29, DJ-1^M26I^: 4.07 ± 0.26; 8 h CCCP 10 μM + 8 h CLQ 1:1000 Mock: 2.31 ± 1.01, wt DJ-1: 2.57 ± 1.48, DJ-1^D149A^: 2.49 ± 0.81, DJ-1^M26I^: 3.31 ± 1.49 (3 < *n* < 7, no variance was observed)]. Data are shown as mean ± SEM. **(I–K)** Mitochondrial extracts were analyzed by Western blotting using the following antibodies: anti-PINK1, anti-LC3, and anti-ATPβ (loading control). The graph represents the quantification of PINK1 **(J)** [PINK1 density, untreated Mock: 0.04 ± 0.03, wt DJ-1: 0.04 ± 0.03, DJ-1^D149A^: 0.04 ± 0.04, DJ-1^M26I^: 0.04 ± 0.03; 8 h CCCP 10 μM Mock: 0.83 ± 0.01, wt DJ-1: 0.81 ± 0.04, DJ-1^D149A^: 0.71 ± 0.04, DJ-1^M26I^: 0.98 ± 0.20 (3 < *n* < 5; no variance was observed)], and LC3 **(K)** expression [LC3II density, 8 h CCCP 10 μM Mock: 0.33 ± 0.02, wt DJ-1: 0.20 ± 0.02, DJ-1^D149A^: 0.46 ± 0.02, DJ-1^M26I^: 0.49 ± 0.02 (*n* = 3; ^∗∗∗^*p* < 0.001; ^∗∗^0.001 < *p* < 0.01; ^∗^0.01 < *p* < 0.05)]. Data are shown as mean ± SEM. In panel **(L)** a representative Western blot on protein ubiquitination is shown and quantified in the corresponding graph **(M)** normalized to GAPDH levels [ubiquitination density, untreated Mock: 1 ± 0.00, wt DJ-1: 1.14 ± 0.01, DJ-1^D149A^: 1.13 ± 0.01, DJ-1^M26I^: 1.40 ± 0.03; 8 h CCCP 10 μM Mock: 1.53 ± 0.01, wt DJ-1: 1.30 ± 0.05, DJ-1^D149A^: 1.21 ± 0.05, DJ-1^M26I^: 0.73 ± 0.05; data are shown as mean ± SEM (*n* = 3; ^∗∗∗^*p* < 0.001; ^∗∗^0.001 < *p* < 0.01; ^∗^0.01 < *p* < 0.05).

Since DJ-1 has been previously implicated in inducing autophagy of damaged organelles (Ariga et al., 2013; Maita et al., 2013), we also assessed the effect of the DJ-1^M26I^ and DJ-1^D149A^ mutants on mitochondrial mass, as well as the efficiency of mitochondrial quality control by targeted autophagy. Image-based analysis of mitochondrial network integrity in cells co-expressing DJ-1^M26I^ and mtGFP did not reveal any visible alterations (**Figures 3A,B**) differently from **Figures 2A,B**. However, quantification of mitochondrially encoded cytochrome c oxidase I (MTCO1) by Western blotting indicated a reduction in protein content both in presence/absence of carbonyl cyanide m-chlorophenyl hydrazone (CCCP) (8 h, 10 μM), in neurons expressing DJ-1^M26I^ compared to wt DJ-1 and DJ-1^D149A^ (**Figures 3C,D**). Even though under resting conditions, mutant DJ-1^D149A^ decreases mitochondrial length (**Figures 2A,B**), both mitochondrial content measured using the MTCO1/β-tubulin ratio (**Figures 3C,D**) and mtDNA number appear to be increased (**Supplementary Figure S2b**). The induction of mitochondrial autophagy (mitophagy), by exposing cells expressing DJ-1^D149A^ and DJ-1^M26I^ to CCCP (**Figures 3C–H**), resulted in a greater reduction of mitochondrial content measured by MTCO-1 and VDAC amount in DJ-1^M26I^ compared to cells expressing wt DJ-1 or DJ-1^D149A^ (**Figures 3C–E,G**). To confirm that this reduction was dependent on mitophagy, we repeated the analysis following inhibition of autophagy flux by CLQ (8 h, 1:1000), which rescued mitochondrial VDAC levels. This suggests that removal of mito-content is mediated by the autophagosome–lysosome pathway (**Figures 3E,F,H**). The involvement of mitophagy mediators in organelle homeostasis was also confirmed by profiling SQSTM1/P62 and LC3 protein expression (**Figures 3F–H**). The degree of PINK1 accumulation and LC3 activation in mitochondria-enriched fractions was then assessed, revealing an increase upon CCCP treatment in cells expressing DJ-1^M26I^ (**Figures 3I–K**). The recruitment of Parkin occurred with the same efficiency for all DJ-1 mutants (data not shown), even though in DJ-1^M26I^ expressing cells the ubiquitination of proteins is far higher under resting conditions (**Figures 3L,M**). However, after treatment with CCCP, ubiquitination was lower than for wt DJ-1 and DJ-1^D149A^. This implies a rapid swing in ubiquitination, which could account for a change in quality control mechanisms in cells expressing DJ-1^M26I^ secondary to the accumulation of PINK1. We therefore postulate that in cells expressing DJ-1^M26I^, an interdependent relationship might exist between SUMOylation and ubiquitination of proteins that could translate into dysregulated mitophagy (**Supplementary Figure S2c**).

### Pattern of DJBP Expression and Intracellular Accumulation During Mitophagy

**FIGURE 4:**
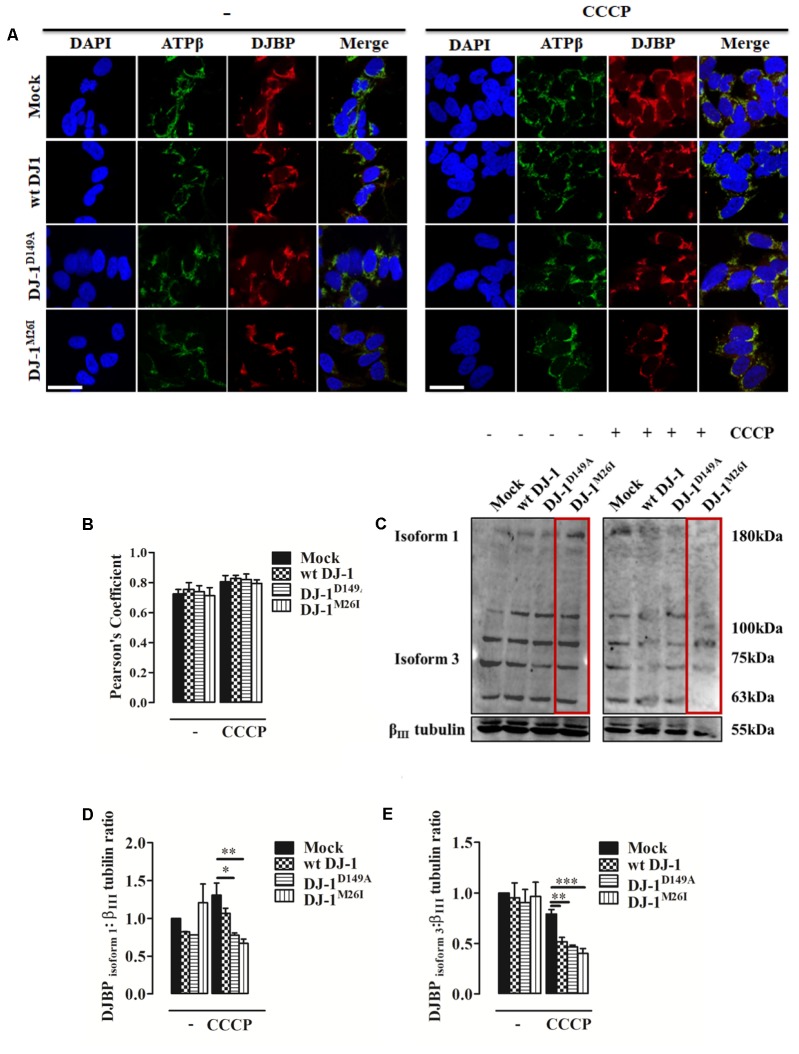
During mitophagy DJBP is recruited to mitochondria. **(A)** Representative immunofluorescence of double staining of DJBP (red) and ATPβ (green) fluorescence in SH-SY5Y cells transfected with FLAG-DJ-1 (wt DJ-1) or mutant FLAG-DJ-1 constructs before and after induction of mitophagy via CCCP (8 h, 10 μM). Scale bar = 10 μm. **(B)** The bar graph shows the quantitative analysis of co-expression levels by Pearson’s coefficient [untreated Mock: 0.72 ± 0.03, wt DJ-1: 0.76 ± 0.04, DJ-1^D149A^: 0.74 ± 0.04, DJ-1^M26I^: 0.71 ± 0.05; 8 h CCCP 10 μM Mock: 0.80 ± 0.04, wt DJ-1: 0.83 ± 0.02, DJ-1^D149A^: 0.82 ± 0.04, DJ-1^M26I^: 0.79 ± 0.02; data are shown as mean ± SEM (*n* = 3; no variance was observed)]. Panel **(C)** shows Western blot analysis of DJBP in total extracts during mitophagy, which reveals protein isoforms, analyzed and quantified in **(D,E)** using anti-DJBP and anti-β_III_ tubulin (loading control) antibodies [DJBP_isoform1_ density, untreated Mock: 1 ± 0.00, wt DJ-1: 0.82 ± 0.002, DJ-1^D149A^: 0.78 ± 0.001, DJ-1^M26I^: 1.21 ± 0.248; 8 h CCCP 10 μM Mock: 1.31 ± 0.16, wt DJ-1: 1.07 ± 0.07, DJ-1^D149A^: 0.78 ± 0.03, DJ-1^M26I^: 0.67 ± 0.06; DJBP isoform 3 density, untreated Mock:1 ± 0.00, wt DJ-1:0.95 ± 0.15, DJ-1^D149A^: 0.90 ± 0.13, DJ-1^M26I^: 0.96 ± 0.14; 8 h CCCP 10 μM Mock: 0.79 ± 0.04, wt DJ-1: 0.52 ± 0.04, DJ-1^D149A^: 0.47 ± 0.02, DJ-1^M26I^: 0.40 ± 0.05; data are shown as mean ± SEM (*n* = 3; ^∗∗∗^*p* < 0.001; ^∗∗^0.001 < *p* < 0.01; ^∗^0.01 < *p* < 0.05)].

An initial analysis of DJBP localization using immuno-fluorescence with a polyclonal anti-DJBP antibody demonstrated that following CCCP treatment, DJBP translocates to mitochondria irrespective of the DJ-1 mutant co-expressed (**Figures 4A,B**). This was further explored through Western blotting analysis of whole-cell lysates (**Figures 4C–E**). DJBP is found in various isoforms (**Supplementary Figure S3a**) and we noticed that during mitophagy the levels of the DJBP long isoform 1 (180 kDa) and isoform 3 (70 kDa) are reduced in cells overexpressing DJ-1 and DJ-1^M26I^ (**Figures 4C–E**). Mitochondrial fractions were also analyzed in detail (**Supplementary Figures S3b,c**) demonstrating that: (i) isoform 3 of DJBP (70 kDa) translocates from the cytosol to mitochondria during mitophagy although this effect was decreased in the presence of DJ-1^M26I^. This could be either indicative of: (i) a greater translocation to mitochondria or (ii) a high degree of DJBP degradation on co-expression with DJ-1^M26I^. Interestingly, these data suggest that even though isoform 3 of DJBP is missing the N-terminal 931 aa, it may contribute to the mitophagic response or act as a mitophagy substrate, driving hyper-activation of the process overall. In addition, the 100 and 63 kDa DJBP isoforms were markedly reduced in whole-cell lysate of cells expressing DJ-1^M26I^ (**Figure 4C**) suggesting that these may also activate mitophagy. By contrast, expression of the DJ-1^D149A^ mutation – which disrupts the DJ-1/DJBP interaction – is likely to recruit less DJBP to damaged mitochondria, thus reducing the availability of this important interactor.

### Dopamine Induces Cell Death by Mitophagy Dysregulation

**FIGURE 5:**
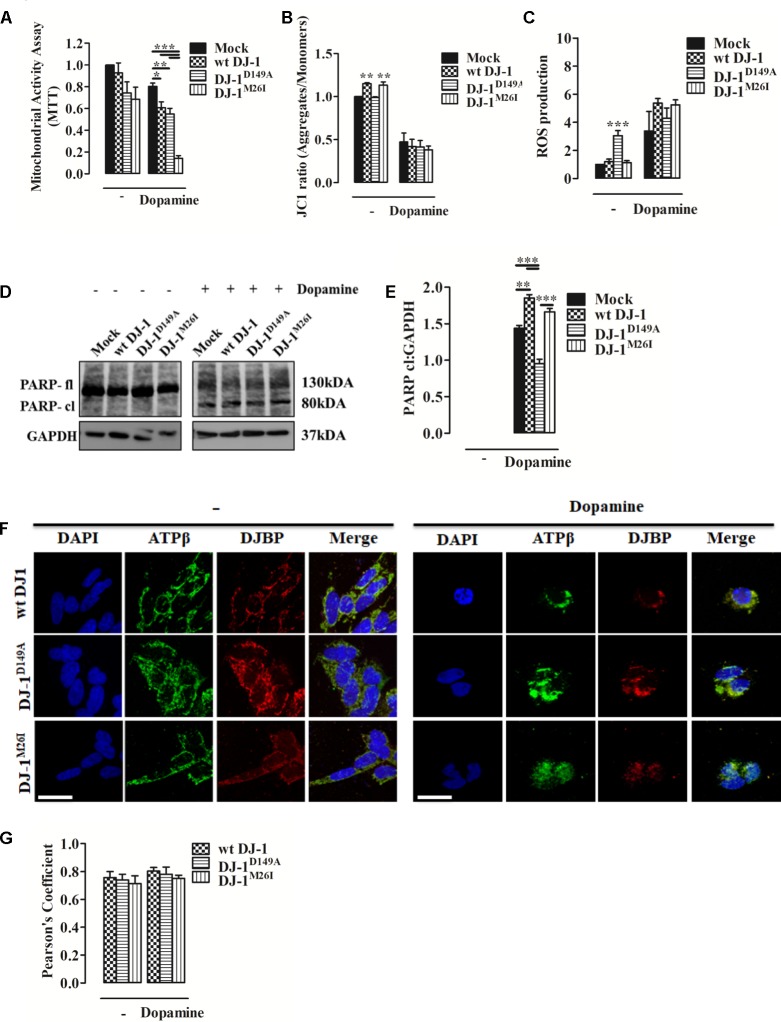
Dopamine-triggered cell death is mediated by dysregulated mitophagy unveiling a specific pathomechanism for DJ-1^M26I^. **(A)** Effect of dopamine on mitochondrial activity. SH-SY5Y cells were transiently co-transfected with FLAG-DJ-1 (wt DJ-1) or mutant FLAG-DJ-1 and YFP constructs. Graph shows mitochondrial activity in cells incubated with 300 μM dopamine for 24 h [MTT, untreated Mock: 1 ± 0.00, wt DJ-1: 0.93 ± 0.09, DJ-1^D149A^: 0.74 ± 0.10, DJ-1^M26I^: 0.68 ± 0.11; 24 h dopamine 300 μM Mock: 0.80 ± 0.03, wt DJ-1: 0.61 ± 0.05, DJ-1^D149A^: 0.55 ± 0.05, DJ-1^M26I^: 0.14 ± 0.02; data are shown as mean ± SEM (*n* = 4; ^∗∗∗^*p* < 0.001; ^∗∗^0.001 < *p* < 0.01; ^∗^0.01 < *p* < 0.05)]. **(B)** Effect of dopamine on the loss of mitochondrial membrane potential. SH-SY5Y cells were transiently transfected with FLAG-DJ-1 (wt DJ-1) or mutant FLAG-DJ-1. At 24 h after transfection, cells were treated with 300 μM dopamine for 24 h and stained with 1 μM JC1. The mean JC1 fluorescence intensity was detected using FACS analysis [JC1 ratio, untreated Mock: 1 ± 0.00, wt DJ-1: 1.15 ± 0.01, DJ-1^D149A^: 0.99 ± 0.01, DJ-1^M26I^: 1.13 ± 0.04; 24 h dopamine 300 μM Mock: 0.47 ± 0.10, wt DJ-1: 0.42 ± 0.08, DJ-1^D149A^: 0.41 ± 0.07, DJ-1^M26I^: 0.38 ± 0.05; data are shown as mean ± SEM (*n* = 3; ^∗∗^0.001 < *p* < 0.01)]. **(C)** Effect of dopamine on mitochondrial O^2-^ production. SH-SY5Y cells, transiently transfected with FLAG-DJ-1 (wt DJ-1) or mutant FLAG-DJ-1, were treated with 300 μM dopamine for 24 h and stained with the fluorescent probe MitoSOX 5 μM. The mean MitoSOX fluorescence intensity was detected using FACS analysis [ROS production, untreated Mock: 1 ± 0.00, wt DJ-1: 1.20 ± 0.18, DJ-1^D149A^: 3.05 ± 0.36, DJ-1^M26I^: 1.13 ± 0.14; 24 h dopamine 300 μM Mock: 3.38 ± 1.38, wt DJ-1: 5.28 ± 0.25, DJ-1^D149A^: 4.62 ± 0.62, DJ-1^M26I^: 4.96 ± 0.38; data are shown as mean ± SEM (3 < *n* < 6; ^∗∗∗^*p* < 0.001). **(D,E)** SH-SY5Y cells transiently co-transfected with FLAG-DJ-1 (wt DJ-1) or mutant FLAG-DJ-1 and YFP constructs were treated with 300 μM dopamine for 24 h. Total extracts were then analyzed by Western blotting **(D)** using anti-PARP and anti-GAPDH (loading control) antibodies. **(E)** Graphical quantification of cleaved PARP expression. Data were normalized on the basis of GAPDH levels [PARP cl density, 24 h dopamine 300 μM Mock: 1.44 ± 0.04; wt DJ-1: 1.85 ± 0.05, DJ-1^D149A^: 0.95 ± 0.06, DJ-1^M26I^: 1.66 ± 0.05 (*n* = 3; ^∗∗∗^*p* < 0.001; ^∗∗^0.001 < *p* < 0.01)]. **(F,G)** DJBP expression after dopamine treatment. Representative image for double staining of DJBP (red) and ATPβ (green) immunofluorescence in SH-SY5Y cells transfected with FLAG-DJ-1 (wt DJ-1) or mutant FLAG-DJ-1 constructs. Scale bar = 10 μm. **(G)** The bar graph shows the quantitative analysis of co-expression levels by Pearson’s coefficient [untreated wt DJ-1: 0.76 ± 0.04, DJ-1^D149A^: 0.74 ± 0.04, DJ-1^M26I^: 0.71 ± 0.05; 24 h dopamine 300 μM wt DJ-1: 0.80 ± 0.03, DJ-1^D149A^: 0.78 ± 0.05, DJ-1^M26I^: 0.75 ± 0.02; data are shown as mean ± SEM (*n* = 3; no variance was observed)].

Dopamine can inhibit Complex I of the mitochondrial respiratory system, thus disrupting mitochondrial activity (Brenner-Lavie et al., 2008). Moreover, it causes extensive loss of cell viability, increased ROS production, and over-expression and accumulation of intracellular α-synuclein (Banerjee et al., 2014). When we analyzed metabolic toxicity associated with mitochondria in dopamine-treated SH-SY5Y cells, we observed a significant reduction that was greater in cells expressing DJ-1^M26I^ (**Figure 5A**). We also investigated Δψ_m_ levels and ROS accumulation finding that after dopamine administration the Δψ_m_ drops sharply, paralleled by an increase in mitochondrial ROS levels, regardless of the DJ-1 mutant tested (**Figures 5B,C**). Notably, the accumulation of ROS in response to dopamine leads to PARP activation that did not differ between the DJ-1 mutants tested (**Figures 5D,E**). Interestingly, the mitochondrial recruitment of DJBP also takes place during dopamine treatment (**Figures 5F,G**). Thus, exploitation of dysregulated mitophagy appears to be the mechanism by which dopamine exhibits greater toxicity in the cells expressing DJ-1^M26I^. Since cells expressing DJ-1^M26I^ are less sensitive to oxidation-induced cell death (**Figure 2H**), individual DJ-1 mutants appear to confer different cellular weaknesses, which only manifest when different insults are faced.

## Discussion

Mitochondria are hubs for the wellbeing of mammalian cells, particularly in post-mitotic cells, such as neurons, which heavily rely on their fitness for energy balance, signaling regulation, and structural preservation. Neurodegenerative conditions therefore associate with defective mitochondrial physiology. Impairment in the mechanisms of organelle quality control such as mitophagy are possible predictable targets to prevent reverse, or at least mitigate, the pathomechanism onset of neuronal death (East and Campanella, 2016; Martinez-Vicente, 2017; Matic et al., 2017). Defective mitochondria underlies the development of neuropathology, by increasing the susceptibility of neurons to auto/paracrine stimulations that will ultimately damage them irreparably, and lead to irreversible demise (Dias et al., 2013). Mitochondria are very responsive organelles, highly sensitive to alterations of the molecular pathways that reduce the efficiency of mitophagy, as the ones involved in neurological conditions (e.g., the anti-mitophagy protein TSPO) (Gatliff et al., 2014). In PD, dopamine-secreting cells undergo cell death (Dauer and Przedborski, 2003), but there is still substantial speculation on the actual mechanisms underlying the process of demise, and how these relate to the small proportion of PD cases with a genetic origin (Dauer and Przedborski, 2003). In the attempt to enlighten the genetic contribution to idiopathic PD conditions, we focused on the effect of specific mutations affecting DJ-1, by measuring their outcome on: (a) DJ-1-accessory protein interactions, (b) mitochondrial morphology, (c) Ca^2+^ signaling, and (d) quality control via targeted autophagy, in order to reveal mutation-specific effects as well as susceptibility to damaging factors such as Ca^2+^ accumulation or redox events. The DJ-1^D149A^ mutation (which is one of four DJ-1 mutations that specifically disrupts the interaction between DJ-1 and DJBP) impairs mitochondrial Ca^2+^ dynamics, revealing an essential buffering function of DJBP (**Figures 2C,D**). This is likely to be instrumental under conditions of cellular stress, since cells that have disrupted DJ-1/DJBP interactions were shown to be susceptible to oxidative stress as well as Ca^2+^-dependent mechanisms of cell death (**Figures 2H,I**). This finding is in line with previous work (Duchen, 2012) demonstrating that the cellular capacity for buffering mitochondrial Ca^2+^ is essential for evading neuronal damage. However, our analysis also revealed that the loss of the DJ-1/DJBP interaction was largely irrelevant to mitophagy (**Figures 3C–M**).

By contrast, the mutation DJ-1^M26I^ resulted in an aberrant mitophagy response (**Figures 3C–M**). DJBP and DJ-1 are both relocalized to the mitochondria during treatment with CCCP, a widely adopted inducer of mitophagy (**Supplementary Figure S3b**; Georgakopoulos et al., 2017), suggesting that DJBP is also involved in mitophagy. However, the degree of ubiquitination of mitochondrial proteins was far greater when the DJ-1 interaction with SUMO-1 was impaired (**Figures 3L,M,O**). The ubiquitination of mitochondrial proteins represents an essential step of commitment to autophagic selection, against which we can score mitochondria undergoing mitophagy and those that are likely to evade the process (Matic et al., 2017). Upon mitophagy induction, cells over-expressing DJ-1^M26I^ show an increased turnover of mitochondrial proteins, as suggested by the analysis of the mtDNA-encoded protein MTCO1 (**Figures 3C,D**) and DNA content (**Supplementary Figure S2b**) at resting conditions. Mitochondrial proteins were retained during the full depolarization of the organelles (**Figures 3C–E,G**), despite the observation that mitochondrial accumulation of LC3II and PINK1 proteins is of equal magnitude (**Figures 3I–K**). These results led us to speculate that by impairing the interaction between DJ-1 and SUMO-1, the DJ-1^M26I^ mutation is likely to favor ubiquitin-mediated mechanisms of degradation, thus resulting in over-active mitophagy.

Interestingly, high dopamine doses amplify mitophagy, causing greater susceptibility to metabolism impairment in the DJ-1^M26I^ mutant (**Figure 5A**). Neuronal cells expressing this mutant were nonetheless protected from oxidative stress-induced cell death (**Figure 2H**), which could be a consequence of the upregulated mitophagy. In fact, removal of mitochondria would reduce the organelle contribution to stimuli exploiting the accumulation of free radicals. Cells over-expressing wt DJ-1 are therefore protected from this type of demise, likely via the core-detoxifying function of the protein itself, as revealed by the analysis of organelle morphology (**Figures 2F,G**). Parameters examined in the presence of excessive doses of dopamine, such as dissipation of Δψ_m_, accumulation of mitochondrial ROS, and PARP cleavage (Abeti et al., 2011), respond similarly regardless of the DJ-1 mutant tested (**Figures 5B–E**). These results are peculiar, given that the DJ-1^D149A^ mutation results in an underlying increased level of free radicals under stress (H_2_O_2_) (**Figure 2E**). It could be that the lack of interaction with DJBP promotes a protective outcome at cytosolic level rather than in mitochondria, whereas a portion of DJ-1 ameliorates the function of PARP resulting in a normalization of the response during dopamine treatment (**Figures 5D,E**). Other mechanisms are likely to come into play when the inducer of death is different, such as during acute exposure to H_2_O_2_ (**Figure 2H**) or ceramide (**Figure 2I**). Whether the trafficking of DJ-1 and DJBP represents a factor in the process of mitochondrial selection as well as toward dopamine susceptibility remains debatable since both their degree of recruitment to the organelle (**Figures 4A,B**) and their specific degradation are unchanged (**Figures 4C–E**). It must be noted that the high degree of mitochondrial turnover as well as the tools for DJBP detection might represent a bias in the assessment of both these processes.

Nevertheless, taken together, our findings demonstrate that loss of interaction between DJ-1 and DJBP is a common pathomechanism of DJ-1 mutations, and leads to aberrant Ca^2+^ signaling that is deleterious in conditions of excessive, non-physiological, mobilization of Ca^2+^ as well as during redox stress (H_2_O_2_). The loss of interaction between DJ-1 and SUMO-1 is a second pathomechanism that does not affect these processes, but is nonetheless pivotal in increasing susceptibility to CCCP and dopamine-driven damage, likely by exploiting the increase in mitophagy. In conclusion, we propose that different DJ-1 mutations produce selective mitochondrial pathologies, resulting in susceptibility to different stressors. This would in turn suggest that cases associated with genetically defined DJ-1 mutations should be treated in accordance with this overall pathophysiological context, including the environmental stressors which could accelerate neuronal death. In our view, this investigation confirms the existing interdependence between genetic background and stressors in PD, as well as indicating how genetic mutations in *PARK7* represent an essential, but not sufficient, prerequisite for the disease to manifest. Taken together, our results suggest that there is more to be learnt about specific cellular pathways underlying PD and once again place mitochondrial dysfunction center stage in disease pathogenesis.

## Author Contributions

KH, RH, and MC conceived, designed, and coordinated the project. MC, DS, VdB, RH, and AR performed the experiments and ran the analysis, to which LR, CF, and CR contributed. All authors critically reviewed and approved the manuscript.

## Conflict of Interest Statement

The authors declare that the research was conducted in the absence of any commercial or financial relationships that could be construed as a potential conflict of interest.

## Supplementary Material

**FIGURE S1 |** Bioinformatic analysis of DJBP mitochondrial targeting sequence and validation of DJ-1 mutant expression. **(a)** Results from YTH β-galactosidase filter assays measuring the effect of DJ-1 mutations on DJ-1 dimerization (see e.g., **Figure 1a**). The intensity of the detected interaction monitored by β-galactosidase activity in the presence of the colorless compound X-gal, which turns blue in the presence of this enzyme, is denoted as follows: (—), no interaction; (+++), strong interaction. **(b)** Potential mitochondrial-targeting motifs in DJBP. Top panel: a potential R-10 motif in the 1501 amino acid isoform of DJBP compared to similar motifs in human ornithine transcarbamylase (hOTC) and human malate dehydrogenase (hMDH2). Lower panel: identification of four R-2 motifs within the N-terminal sequence in the 1501 amino acid isoform of DJBP compared to similar motifs in aspartate aminotransferase 2 (hAAT2) and succinyl-CoA synthetase alpha subunit (hSCSA). **(c)** SH-SY5Y cells were transiently co-transfected with pcDNA3.1(+)/FLAG and YFP construct using calcium phosphate method. Green fluorescence is an indicator that the FLAG-DJ-1-containing transcript is being expressed. Scale bar = 10 μm. **(d)** Analysis of steady-state levels of DJ-1 mutants. Whole-cell extracts from SH-SY5Y cells co-transfected with FLAG-wtDJ-1 or mutant FLAG-DJ-1 and YFP construct was analyzed by Western blot with anti-FLAG and anti-DJ-1 antibody. Upper band: exogenous FLAG tagged DJ1; Lower band: endogenous DJ-1. **(e)** The graph represents the quantification of endogenous and FLAG-DJ-1 normalized on the basis of GAPDH levels (*n* = 3).

**FIGURE S2 |** Mitochondrial O^2-^ production, DNA copy number, and proteins processing. **(a)** Mitochondrial O^2-^ was evaluated in SH-SY5Y cells transiently transfected with FLAG-DJ-1 (wt DJ-1) or mutant FLAG-DJ-1 using the fluorescent probe MitoSOX (5 μM). The graph represents the mean of MitoSOX fluorescence intensity detected by FACS analysis [ROS production, untreated Mock: 1 ± 0.00, wt DJ-1: 1.20 ± 0.18, DJ-1^D149A^: 3.05 ± 0.36, DJ-1^M26I^: 1.13 ± 0.14; data are shown as mean ± SEM (*n* = 3; ^∗∗∗^*p* < 0.001)]. **(b)** Quantification of mtDNA. The mtDNA copy number/cell was evaluated in SH-SY5Y transiently transfected with FLAG-DJ-1 (wt DJ-1) or mutant FLAG-DJ-1 constructs [untreated 1 ± 0.00, wt DJ-1: 1.18 ± 0.07, DJ-1^D149A^: 1.38 ± 0.15, DJ-1^M26I^: 1.14 ± 0.02; data are shown as mean ± SEM (*n* = 4; ^∗^0.01 < *p* < 0.05)]. **(c)** Representative Western blot showing the balance in protein ubiquitination and sumoylation in SH-SY5Y cells expressing the anti-mitophagy protein TSPO, which de-ubiquitinates mitochondria.

**FIGURE S3 |** DJBP sequences alignment and pattern during mitophagy. **(a)** Isoforms of human DJBP. The C-term portion contains the interaction domain with DJ-1 in position 1303–1501. Isoform 1 is the canonical sequence of DJBP. Isoform 2 is missing amino acids 1–152. Isoform 3 is missing amino acids 1–931. Isoform 4 is missing amino acids 1–1139 and has glutamic acid instead of methionine at position 1140. Isoform 5 is missing amino acids 1250–1501 and has an alternative sequence at position 1217–1249. Isoform 6 is missing amino acids 421–1501 and has an alternative amino acid sequence at position 418–420 (KPD > VRF). **(b)** Pattern of DJBP isoform expression after CCCP treatment (8 h, 10 μM) in mitochondrial fractions of SH-SY5Y cells expressing DJ-1 mutants with the cytosolic counterpart is reported in **(c)**.

**TABLE S1 |** Oligonucleotide primers used for cloning. Restriction enzyme recognition sites within primers are indicated by underlining.

**TABLE S2 |** Primer sets used for site-directed mutagenesis of DJ-1. The forward primer is shown above the reverse primer for each mutant. The underlined sequence indicates the codon containing the nucleotide change.
