# Supplementary figures and images for: Distinct Mechanisms of Pathogenic DJ-1 Mutations in Mitochondrial Quality Control
Source: Front Mol Neurosci. 2018 Mar 15;11:68. doi: 10.3389/fnmol.2018.00068 (PMC5862874; doi:10.3389/fnmol.2018.00068)

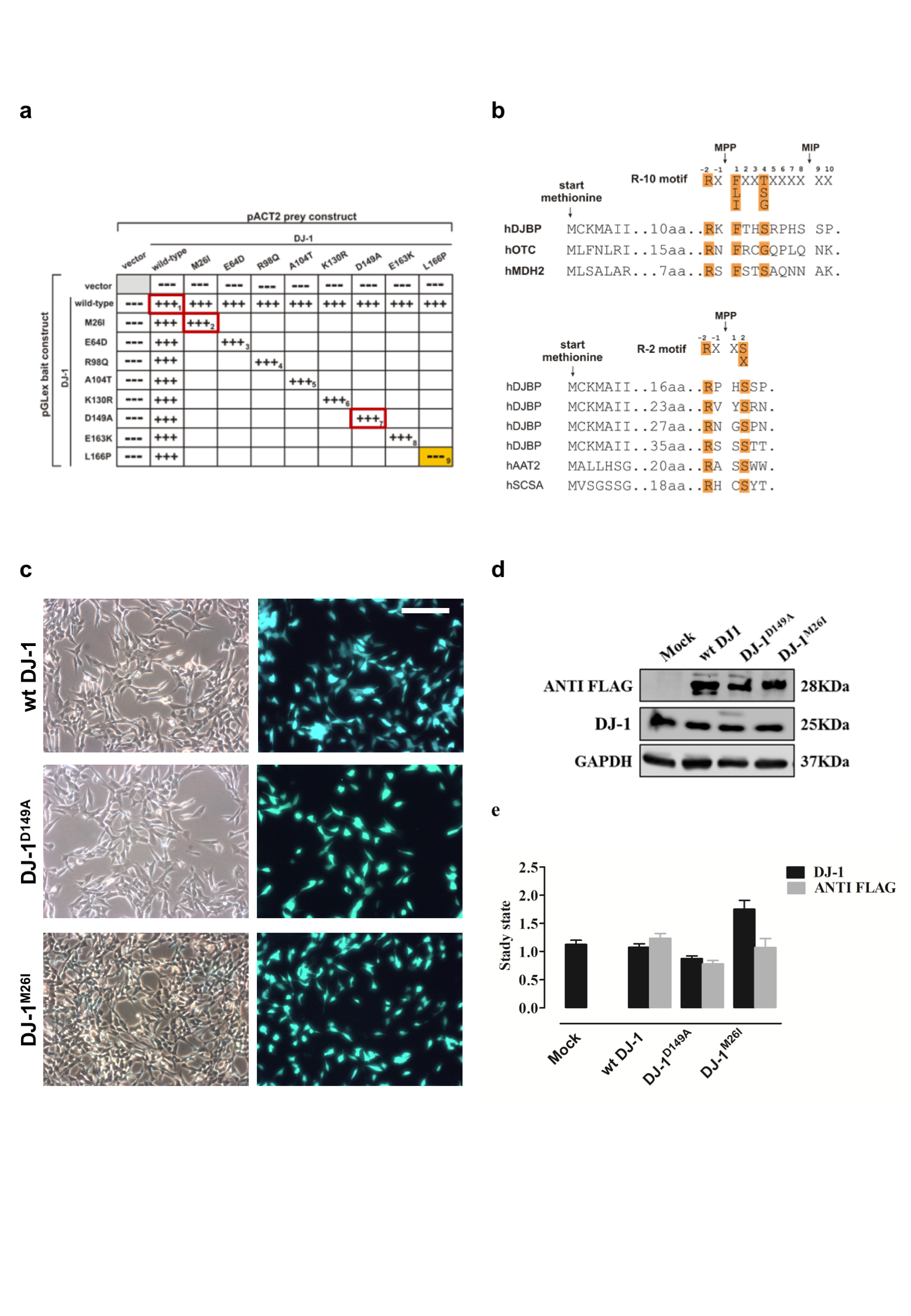

Supplement: Supplementary file 1 [file Image_1.TIF]

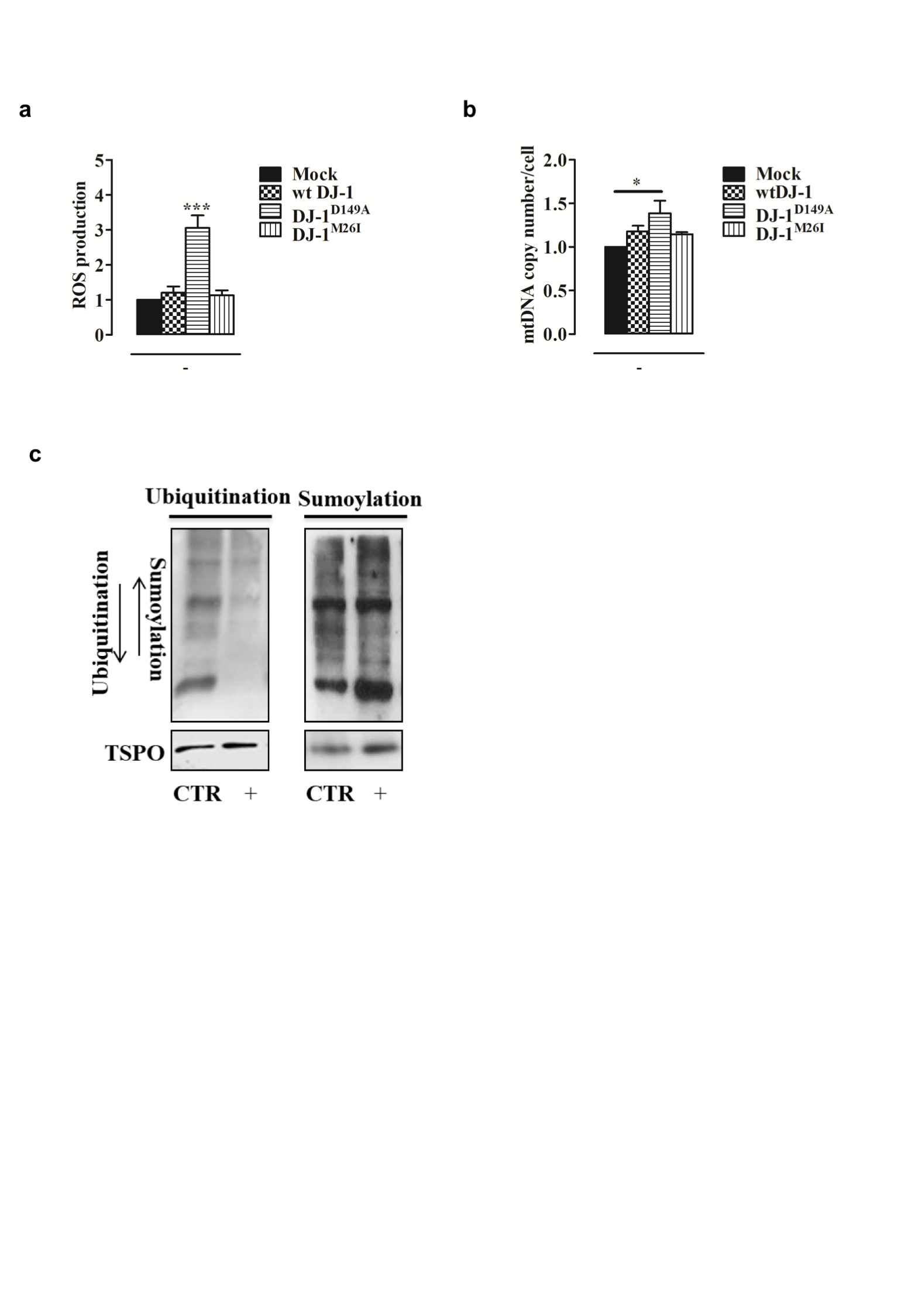

Supplement: Supplementary file 2 [file Image_2.TIF]

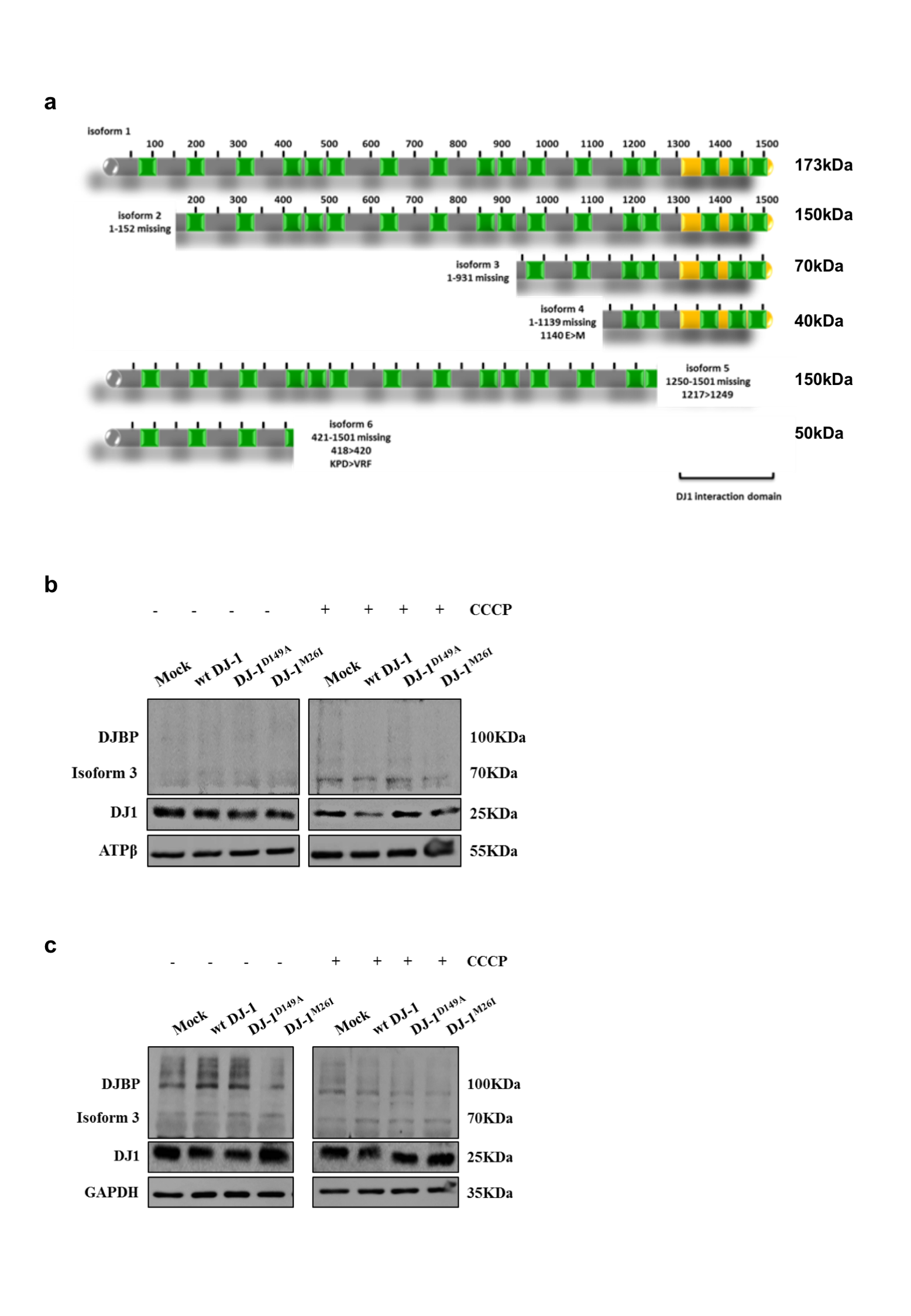

Supplement: Supplementary file 3 [file Image_3.TIF]
