# Supplementary material for: Distinct Mechanisms of Pathogenic DJ-1 Mutations in Mitochondrial Quality Control
Source: Front Mol Neurosci. 2018 Mar 15;11:68. doi: 10.3389/fnmol.2018.00068 (PMC5862874; doi:10.3389/fnmol.2018.00068)
Supplement: Supplementary file 4 [file Data_Sheet_1.docx]

| **Primer name** | **Direction** | **Primer sequence** | **Enzyme** |
| --- | --- | --- | --- |
| FLAG-DJ-1 | F | 5'-aacgaattcatggcttccaaaagagc-3' | *Eco*RI |
| FLAG-DJ-1 | R | 5'-ttcctcgagctagtctttaagaacaa-3' | *Xho*I |
| pACT2-DJ-1 | F | 5'-gagggatccatggcttccaaaagagc-3' | *NcoI* |
| pACT2-DJ-1 | R | 5'-ttcctcgagctagtctttaagaacaa-3' | *Xho*I |
| pACT2-DJBP1 | F | 5'-actccatgggtcattttacaaagcc-3' | *Nco*I |
| pACT2-DJBP2 | R | 5'-cagctcgagctactggaggaatgcc-3' | *Xho*I |
| pACT2- SUMO-1 | F | 5'-tcaccatggctgaccaggaggcaaa-3' | *Nco*I |
| pACT2- SUMO-1 | R | 5'-aaactcgagctaaactgttgaatga-3' | *Xho*I |
| pACT2-UBC9 | F | 5'-tgaccatggcggggatcgccctcag-3' | *Nco*I |
| pACT2-UBC9 | R | 5'-ggtctcgagttatgagggcgcaaac-3' | *Xho*I |
| pACT2-DAXX | F | 5'-tttggatccctatggccaccgctaacagca-3' | *Bam*HI |
| pACT2-DAXX | R | 5'-gggctcgagctaatcagagtctgagagcac-3' | *Xho*I |
| pEGFPN-DJBP | F | 5'–tctctcgaggccaccatgtgcaaaatggcg–3' | *Xho*I |
| pEGFPN-DJBP_31 | R | 5'-gaaggatccctacacggtgaagaatggggt-3' | *Bam*HI |
| pEGFPN-DJBP_48 | R | 5'-gcaggatccgtggttgaagaagatctgaac-3' | *Bam*HI |
| myc-DJBP | F | 5'-ttcggatccatgggtcattttacaaa-3' | *Bam*HI |
| myc-DJBP | R | 5'-cagtctagactactggaggaatgccc-3' | *Xba*I |
| DsRed-MITO | F | 5'–gtagaattcgccaccatgtccgtcctgac-3' | *Eco*RI |
| DsRed-MITO | R | 5'-tccggatccaacgaatggatcttggcgcg-3' | *Bam*HI |

**Supplementary Table 1. Oligonucleotide primers used for cloning.** Restriction enzyme recognition sites within primers are indicated by underlining.

| **DJ-1 mutant** | **Mutagenesis primer sequence** |
| --- | --- |
| **M26I** | 5'-cctgtagatgtcataaggcgagctggg-3'  5'-cccagctcgccttatgacatctacagg-3' |
| **E64D** | 5'-gatgcaaaaaaagatggaccatatgat-3' 5'-atcatatggtccatctttttttgcatc-3' |
| **R98Q** | 5'-gagcaggaaaaccagaagggcctgata-3'  5'-tatcaggcccttctggttttcctgctc-3' |
| **A104T** | 5'-ggcctgatagccaccatctgtgcaggt-3'  5'-acctgcacagatggtggctatcaggcc-3' |
| **D149A** | 5'-cgtgtggaaaaagccggcctgattctt-3'  5'-aagaatcaggccggctttttccacacg-3' |
| **E163K** | 5'-gggaccagcttcaagtttgcgcttgca-3'  5'-ccctggtcgaagttcaaacgcgaacgt-3' |
| **L166P** | 5'-ttcgagtttgcgcctgcaattgttgaa-3'  5'-ttcaacaattgcaggcgcaaactcgaa-3' |

**Supplementary Table 2. Primer sets used for site-directed mutagenesis of DJ-1.** The forward primer is shown above the reverse primer for each mutant. The underlined sequence indicates the codon containing the nucleotide change.
